# The characteristics of blood transfusion and analysis of preoperative factors associated with intraoperative blood transfusion in congenital heart surgery: a case–control study

**DOI:** 10.1186/s13019-022-02068-2

**Published:** 2022-12-24

**Authors:** Ming-wei Yin, Bao-hai Chen, Xue-jun Chen, Tao Zhang, Jie Jin, Jun Xu

**Affiliations:** 1 Blood Transfusion Department, The Children’s Hospital, Zhejiang University School of Medicine, National Clinical Research Center for Child Health, No. 3333, Binsheng Rd, Binjiang District, Hangzhou, 310052 Zhejiang Province People’s Republic of China; 2 Information Center, The Children’s Hospital, Zhejiang University School of Medicine, National Clinical Research Center for Child Health, Hangzhou, Zhejiang Province People’s Republic of China; 3 Blood Transfusion Department, Tai’an City Central Hospital, Taian, Shandong Province People’s Republic of China; 4 Cardiac Surgery, The Children’s Hospital, Zhejiang University School of Medicine, National Clinical Research Center for Child Health, Hangzhou, Zhejiang Province People’s Republic of China

**Keywords:** Congenital heart surgery, Preoperative factors, Intraoperative blood transfusion

## Abstract

**Purpose:**

Blood transfusion is a common and life-saving procedure in congenital heart surgery (CHS), and it is critical for patients to identify risk factors prior to surgery. Our objective is to conduct an analysis of the preoperative factors that influence blood use during CHS and to offer guidance on preoperative blood preparation.

**Methods:**

A total of 1550 cases were retrospectively analyzed in our institution between May 2019 and June 2020. We determined whether to employ red blood cells (RBCs), platelets, and plasma as dependent variables; we treated the data from characteristics and laboratory tests as binary data, except for the Risk Adjustment for Congenital Heart Surgery (RACHS) methods as multinomial data, and finally taken into binary logistic regression analysis.

**Results:**

The total amounts of transfused RBCs, platelets, and plasma were 850.5 U (N = 713, 46%), 159 U (N = 21, 1.4%), and 1374.2 U (N = 953, 61.5%), respectively. Multivariate analysis found age (OR 0.142, 95% CI 0.099–0.203, *P* < 0.001), weight (0.170, 0.111–0.262, *P* < 0.001) RACHS method (RACHS2 vs. RACHS1, 3.444, 2.521–4.704, *P* < 0.001; RACHS3 vs. RACHS1, 9.333, 4.731–18.412, *P* < 0.001; RACHS4 vs. RACHS1, 31.327, 2.916–336.546, *P* = 0.004), and hemoglobin (0.524, 0.315–0.871, *P* = 0.013) to be independent risk predictors of RBC transfused volume; age (9.911, 1.008–97.417, *P* = 0.049), weight (0.029, 0.003–0.300, *P* = 0.029), RACHS method (RACHS3 vs. RACHS1, 13.001, 2.482–68.112, *P* = 0.002; RACHS4 vs. RACHS1, 59.748, 6.351–562.115, *P* < 0.001) to be platelets; and age (0.488, 0.352–0.676, *P* < 0.001), weight (0.252, 0.164–0.386, *P* < 0.001), RACHS method (RACHS2 vs. RACHS1, 2.931, 2.283–3.764, *P* < 0.001; RACHS3 vs. RACHS1, 10.754, 4.751–24.342, *P* < 0.001), APTT (1.628, 1.058–2.503, *P* = 0.027), and PT (2.174, 1.065–4.435, *P* = 0.033) to be plasma.

**Conclusion:**

Although patients' age, weight, routine blood test, coagulation function, and protein levels should all be considered for preparing blood before CHS, the RACHS method is the most important factor influencing intraoperative blood transfused volume and should be considered first in clinical blood preparation.

**Supplementary Information:**

The online version contains supplementary material available at 10.1186/s13019-022-02068-2.

## Introduction

Congenital heart defects (CHDs), also known as congenital heart disease, are birth disorders in the structure of the heart or major vessels; the symptoms can range from nonexistent to life-threatening [1]. CHDs are the most common birth defect and the main cause of birth defect-related deaths [2], and surgery was the primary treatment approach, which included both on-pump and off-pump bypass operations. Off-pump bypass surgery usually does not require open-heart surgeries, but cardiopulmonary bypass surgery (CPB) is always required for cardiac internal malformation surgery, which requires greater blood use than the former [3]. Furthermore, CPB-required congenital heart surgery (CHS) is frequently complicated by coagulopathy, which can result in excessive hemorrhage and blood transfusion [3, 4]. Allogeneic transfusion is frequently required in cardiac surgery, with a prior study indicating overall transfusion rates in excess of 50% [5]. For many years, various models for predicting transfusion requirements in cardiac surgery patients have been available in adults [6, 7]. Operative procedure, surgeon, age, sex, height, weight, body surface area (BSA), hematocrit, presence or absence of diabetes, and albumin levels were identified as predictive factors. Parr performed a multivariate predictor of blood product use in cardiac surgery and stated that increased age and preoperative creatinine level, low body surface area, preoperative hematocrit, nonelective surgery, lower temperature on bypass, and duration of bypass were associated with an increased risk of transfusion of > 2 units (U) of red blood cells (RBCs) [7]. Williams evaluated demographic and perioperative factors to identify variables associated with perioperative blood loss and blood product transfusions in a prospective cohort study of 548 children undergoing open-heart surgery and found that higher preoperative hematocrit, complex surgery, lower platelet count during cardiopulmonary bypass (CPB), and longer duration of deep hypothermic circulatory arrest were significantly associated with bleeding and transfusion, and younger patient age was especially found to be the variable most significantly associated with bleeding and transfusions [8]. The Risk Adjustment for Congenital Heart Surgery (RACHS) method was created to allow a refined understanding of differences in mortality among patients undergoing CHS, as would typically be encountered within a pediatric population [9], which divides anatomic variation into six groups based on age, kind of operation performed, and hospital mortality [10], and it is rarely reported in analyzing the influencing factors of surgical blood use.

Because of the wider ranges of characteristics in children, there has always been uncertainty in blood transfusion and less research in predicting blood transfusion. Furthermore, due to the tiny total blood volume in children, bloodless prefilling is difficult to accomplish during extracorporeal circulation, making pediatric CPB procedures more dependent on allogeneic blood than adult surgeries [11]. According to our hospital's summary of application for blood preparation, children receiving CHS should have RBCs, platelets, and plasma prepared at the blood transfusion department 1–3 days in advance. Although the prepared dosages of blood products were usually adequate prior to most CHSs, a few of them consumed blood products greater than the prepared, particularly in the circumstances of transfused dosages of RBCs over 2 U, which was a study for adults [12]. Studies have shown that overemphasis on the use of blood during CHS is not conducive to the postoperative mental development of children [13]. As a result, how to prepare blood products reasonably for children treated by CHS, particularly for patients with rare blood types at the same time, is a common but challenging issue confronting heart surgeons, anesthesia perfusionists, and blood transfusion technologists.

## Methods

This study was designed as a retrospective case–control study, and data from 1,550 children who underwent CHS under CPB (S5, Sorin Group Deutschland GmbH, Germany) using modified ultrafiltration (MUF) perfusion techniques were collected in our hospital between May 2019 and June 2020. The demographic data of patients, as well as other laboratory data, were retrieved from the blood transfusion management system, surgical anesthesia system, and laboratory information system (LIS). We obtained all patients' demographic and procedural data, including sex, age, weight, hemoglobin, RBC counts, hematocrit, platelet counts, white blood cell (WBC) counts, partial thromboplastin time (PT), activated partial thromboplastin time (APTT), albumin (Alb), total protein (TP), and RACHS methods, all of which were performed as independent variables, and intraoperative blood product use was used as a dependent variable. The detailed RACHS categories in this study were listed in Table 1. Suspended RBCs, washed RBCs, and concentrated RBCs are collectively referred to as RBCs (the volumes of 1 U RBCs and 1 U washed RBCs are approximately 150 ml and 130 ml, respectively), fresh frozen plasma and frozen plasma are referred to as plasma (1 U = 100 ml), and apheresis and concentrated platelets are referred to as platelets (the volume of 10 U is approximately 250 ml). All blood products are calculated in international standard units (U). Surgical blood transfusions are defined as blood transfusions given during surgery and prior to returning to the ward. The basic information of the patients, the information of surgical blood preparation and blood use records are complete, and the results of the preoperative laboratory examination are completely recorded. Data validation and integrity will be examined by heart surgeons, anesthetic perfusionists, and blood transfusion physicians to optimize data integrity.

**Table 1:** Individual procedures by RACHS category

RACHS category	Procedure	No	Sum	RACHS category	Procedure	No	Sum
RACHS category 1	PDA > 30 d	98	676	RACHS category 4	TA repair	4	8
	Coarctation > 30 d	167			Double switch	2	
	ASD	411			ASO/VSD	2	
RACHS category 2	VSD	668	777	RACHS category 5	TV repositioning in Ebsteins anomaly < 30 d		0
	TOF	56			Truncus/IAA		
	Pulmonary valvotomy or valvuloplasty	38					
	Aortic valvotomy or valvuloplasty > 30 d	11					
	AP Window	4					
RACHS category 3	ASO	16	89	RACHS category 6	Norwood operation		0
	Mitral/Tricuspid valvotomy or valvuloplasty	20			DKS		
	TOF/PA	10					
	Repair of cor triatriatum	8					
	Repair of transitional or complete atrioventricular canal	20					
	DORV repair	5					
	BTS	2					
	PAB	8					

*RACHS* risk adjustment for congenital heart surgery, *PDA* patent ductus arteriosus, *ASD* atrial septal defect, *VSD* ventricular septal defect, *TOF* tetralogy of Fallot, *AP* aortopulmonary, *ASO* arterial switch operation, *PA* pulmonary atresia, *DORV* double outlet right ventricle, *BTS* Blalock–Tuassig shunt, *PAB* pulmonary artery band, *TA* truncus arteriosus, *TV* tricuspid valve, *IAA* interrupted aortic arch, *DKS* Damus Kaye Stancil, *No*. number

All independent variables were developed as categorical variables, and univariate analysis was conducted using the chi-square test. All significant variables were then included in the multivariate binary logistic regression analysis. The RACHS method was used as multinomial data (RACHS1 was used as the indicator). All statistical analyses were performed using IBM SPSS software (SPSS 20.0, IBM Inc., CA, USA). A two-sided *P* value < 0.05 was considered significant.

## Results

### The characteristics of blood transfusion in pediatric CHS under CPB

**Fig. 1:**
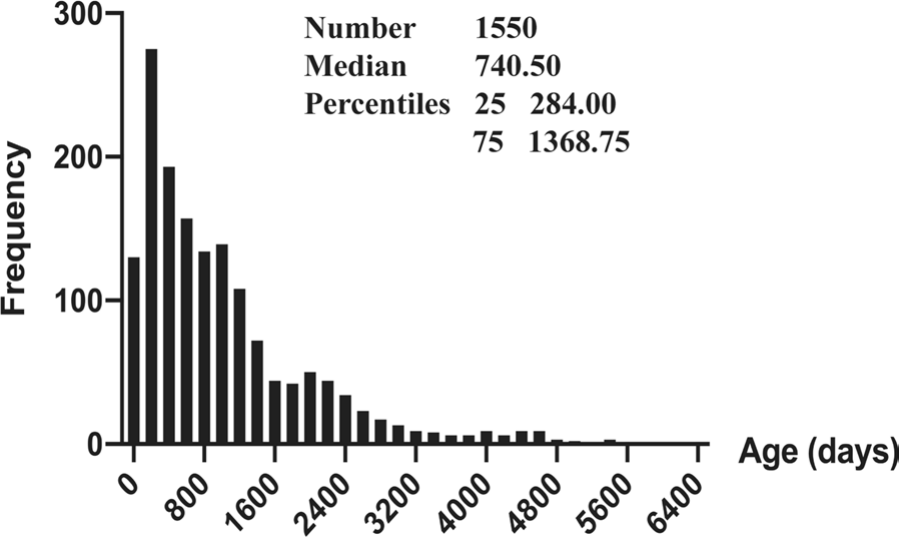
The characteristics of blood transfusion in pediatric CHS under CPB

Among the 1550 cases, 734 male and 816 female cases accounted for 47.4% and 52.6%, respectively. The ages varied from 1 day to 6397 [740.5 (IQR 1084.75) days]. They are classified into two groups based on whether they have used RBCs, platelets, or plasma. Table 2 shows that the total amounts of transfused RBCs, platelets, and plasma were 850.5 U (N = 713, 46%), 159 U (N = 21, 1.4%), 1374.2 U (N = 953, 61.5%), with average volumes of 1.2 U, 7.6 U, 1.4 U, respectively. When the age and frequency association is examined, it is discovered that there is a skewed distribution, with a median of 740.5, 1/4 and 3/4 interquartiles of 284 and 1368.75, respectively (Fig. 1).

**Table 2:** The characteristics of blood transfusion in pediatric CHS under CPB

Blood products	Cases of transfused	Transfused rate (%)	Total transfusion (U)	Mean transfusion (U)
Red blood cells	713	46	850.5	1.19
Platelets	21	1.4	159	7.57
Plasma	953	61.5	1374.2	1.44

### A single-factor study of RBCs, platelets, and plasma transfusion doses in pediatric CHS under CPB

Using chi-square testing, we discovered that weight, age, RACHS method, hemoglobin, RBC counts, Hct, APTT, PT, TP, and ALB all had an effect on the number of RBCs transfused (*P* < 0.05). Weight, age, RACHS method, hemoglobin, RBC counts, PT, TP, and ALB are all variables that may impact platelet confusion (*P* < 0.05). Furthermore, sex, weight, age, RACHS method, hemoglobin, RBC counts, HCT, APTT, PT, and TP were statistically significant between whether plasma was used (*P* < 0.05). The detailed data are shown in Table 3.

**Table 3:** Single factor analysis of RBCs, platelets, and plasma transfused dosages in pediatric CHS under CPB

Factors	Red blood cells				Platelets				Plasma			
	Used	Unused	χ^2^	*P* value	Used	Unused	χ^2^	*P* value	Used	Unused	χ^2^	*P* value
*Gender*												
Male	350	384	1.592	0.207	8	726	0.732	0.392	475	259	6.143	0.013
Female	363	453			13	803			478	338		
*Weight (kg)*												
< 10	490	58	643.271	< 0.001	17	532	19.271	< 0.001	483	65	254.327	< 0.001
≥ 10	223	779			4	996			470	532		
*Age (year)*												
< 2	607	164	661.547	< 0.001	16	757	5.899	0.015	622	149	238.573	< 0.001
≥ 2	106	673			5	772			331	448		
*RACHS method*												
RS1	161	515	255.525	< 0.001	2	674	41.567	< 0.001	277	399	235.573	< 0.001
RS2	478	299			8	769			586	191		
RS3	67	22			8	81			82	7		
RS4	7	1			3	5			8	0		
*Hemoglobin (g/L)*												
< 120	450	253	168.014	< 0.001	1	695	13.865	< 0.001	486	217	31.779	< 0.001
≥ 120	263	584			20	834			467	380		
RBC counts (*10^*12*^*/L)*												
< 3.5	70	2	79.754	< 0.001	6	68	21.477	< 0.001	63	9	21.581	< 0.001
≥ 3.5	643	835			15	1461			890	588		
*HCT (%)*												
< 0.37	490	397	71.311	< 0.001	9	878	1.796	0.18	569	318	6.219	0.013
≥ 0.37	223	440			12	651			384	279		
PLT counts (*10^*9*^*/L)*												
<100	5	1	2.039	0.153	1	7	1.442	0.23	5	1	0.465	0.495
≥ 100	708	836			20	1522			948	596		
WBC counts (*10^*9*^*/L)*												
< 4.3	8	8	0.104	0.747	1	18	0.235	0.628	9	7	0.187	0.665
≥ 4.3	705	829			20	1511			944	590		
*APTT (s)*												
< 34.6	586	778	42.237	< 0.001	16	1341	1.574	0.21	812	547	14.004	< 0.001
≥ 34.6	127	59			5	188			141	50		
*PT (s)*												
< 13.2	649	815	29.603	< 0.001	16	1441	8.985	0.003	880	579	14.331	< 0.001
≥ 13.2	64	22			5	88			73	18		
*TP (g/L)*												
< 60	248	47	212.556	< 0.001	11	305	11.502	0.001	249	65	52.776	< 0.001
≥ 60	465	790			10	1224			704	532		
*ALB (g/L)*												
< 35	37	20	8.521	0.004	5	73	11.976	0.001	39	37	3.489	0.062
≥ 35	676	817			16	1456			914	560		

*CHS* congenital heart surgery, *CPB* cardiopulmonary bypass, *RACHS* risk adjustment for congenital heart surgery, *RBC* red blood cells, *HCT* hematocrit, *PLT* platelets, *WBC* white blood cells, *APTT* activated partial thromboplastin time, *PT* partial thromboplastin time, *Alb* albumin, *TP* total protein

### Multivariate analysis of the RBC, platelet, and plasma transfusion dosages in pediatric CHS under CPB

We determined whether RBCs, platelets, and plasma were used as dependent variables and then gathered all covariates with a *P* < 0.05 and entered them into binary logistic regression analysis using the enter method. We still assessed platelet counts while determining whether platelets should be used based on clinical experiments. The factors associated with RBCs transfusion included four variables: age [OR 0.142 (95% CI 0.099–0.203) *P* < 0.001], weight [OR 0.170 (95% CI 0.111–0.262) *P* < 0.001], RACHS method [RACHS2 vs. RACHS1 OR 3.444 (95% CI 2.521–4.704) *P* < 0.001, RACHS3 vs. RACHS1 OR 9.333 (95% CI 4.731–18.412) *P* < 0.001, RACHS4 vs. RACHS1 OR 31.327 (95% CI 2.916–336.546) *P* = 0.004], and hemoglobin [OR 0.524 (95% CI 0.315–0.871) *P* = 0.013]. The factors associated with platelets transfusion included three variables: age [OR 9.911 (95% CI 1.008–97.417) *P* = 0.049], weight [OR 0.029 (95% CI 0.003–0.300) *P* = 0.029], and RACHS method [RACHS3 vs. RACHS1 OR 13.001 (95% CI 2.482–68.112) *P* = 0.002, RACHS4 vs. RACHS1 OR 59.748 (95% CI 6.351–562.115) *P* < 0.001]. The factors associated with plasma transfusion included five variables: age [OR 0.488 (95% CI 0.352–0.676) *P* < 0.001], weight [OR 0.252 (95% CI 0.164–0.386) *P* < 0.001], RACHS method [RACHS2 vs. RACHS1 OR 2.931 (95% CI 2.283–3.764) *P* < 0.001, RACHS3 vs. RACHS1 OR 10.754 (95% CI 4.751–24.342) *P* < 0.001], APTT [OR 1.628 (95% CI 1.058–2.503) *P* = 0.027], and PT [OR 2.174 (95% CI 1.065–4.435) *P* = 0.033] (detailed in Table 4).

**Table 4:** Multivariate analysis of RBCs, platelets, and plasma transfused dosages in pediatric CHS under CPB

Blood products	Variables	*P*	OR	95%CI	
				Lower	Upper
RBCs	Age	< 0.001	0.142	0.099	0.203
	Weight	< 0.001	0.170	0.111	0.262
	RACHS^a^	< 0.001	3.444	2.521	4.704
	RACHS^b^	< 0.001	9.333	4.731	18.412
	RACHS^c^	0.004	31.327	2.916	336.546
	Hemoglobin	0.013	0.524	0.315	0.871
Platelets	Age	0.049	9.911	1.008	97.417
	Weight	0.003	0.029	0.003	0.300
	RACHS^b^	0.002	13.001	2.482	68.112
	RACHS^c^	< 0.001	59.748	6.351	562.115
Plasma	Age	< 0.001	0.488	0.352	0.676
	Weight	< 0.001	0.252	0.164	0.386
	RACHS^a^	< 0.001	2.931	2.283	3.764
	RACHS^b^	< 0.001	10.754	4.751	24.342
	APTT	0.027	1.628	1.058	2.503
	PT	0.033	2.174	1.065	4.435

*CHS* congenital heart surgery, *CPB* cardiopulmonary bypass, *RACHS* risk adjustment for congenital heart surgery, *APTT* activated partial thromboplastin time, *PT* partial thromboplastin time

^a^RACHS2 versus RACHS1; ^b^RACHS3 versus RACHS1; ^c^RACHS4 versus RACHS1

### Comparisons of RBCs, platelets, and plasma used between different RACHS methods

**Fig. 2:**
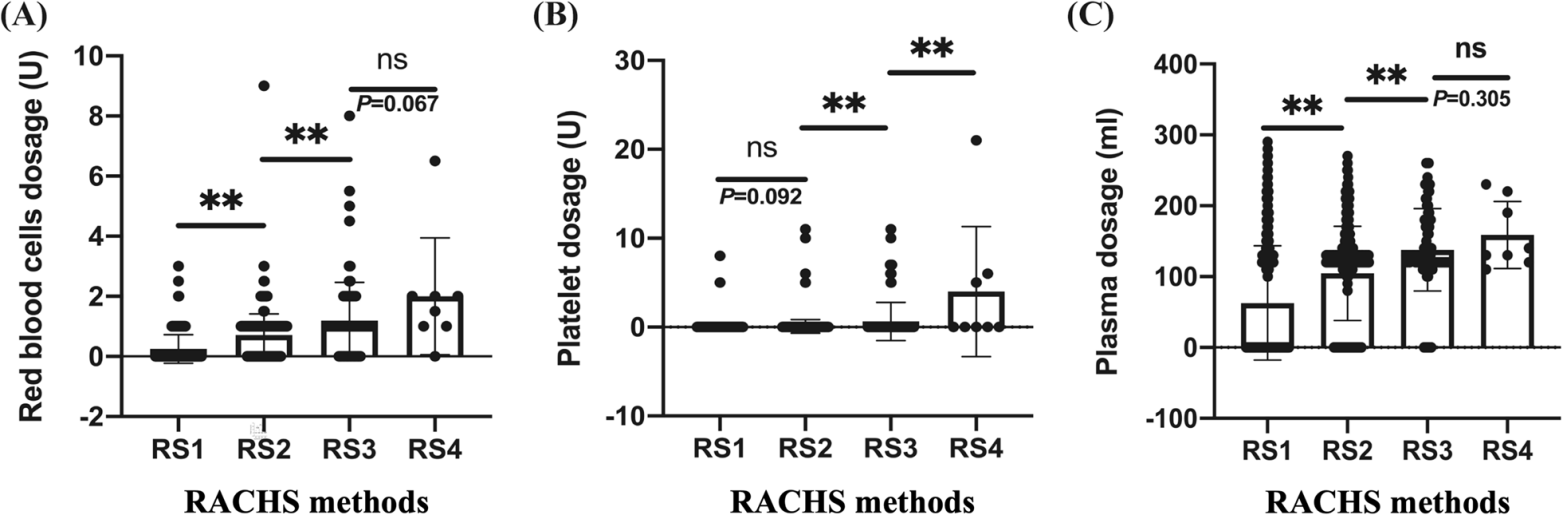
The comparisons of RBCs, platelets, and plasma used across different RACHS methods. **A** The differences in RBC transfused dosage of RS1 versus RS2 and RS2 versus RS3 were statistically significant, but the comparison between RS3 and RS4 was not. **B** There was no significant difference in platelet usage between RS1 and RS2, but there were significant differences in RS2 versus RS3 and RS3 versus RS4. **C** The differences in plasma transfused dosage of RS1 versus RS2 and RS2 versus RS3 were statistically significant, but the comparison between RS3 and RS4 was not. **P < 0.01; ns, no significance

We discovered that the OR values of RACHS methods were the maximum in all variables in RBCs, platelets, and plasma transfusion in the aforementioned results. We compared the transfused dosages in different RACHS methods in different blood products as the outcomes to further reveal the roles of the RACHS method. Among the 1550 instances, 676, 777, 89, and 8 were assigned to the RS1, RS2, RS3, and RS4 categories, respectively. Overall, there has been a tendency that the higher the RACHS grade, the more blood was required. On the transfused RBCs, the comparisons between RS1 versus RS2 and RS2 versus RS3 were statistically significant (*P* < 0.001), but there was no significant difference between RS3 and RS4 (*P* = 0.067). Concerning the platelets consumed, the trend was not exactly the same as that of RBCs; the contrast between RS1 and RS2 was not statistically significant (*P* = 0.092), while those in RS2 versus RS3 and RS3 versus RS4 were significantly different (*P* < 0.001). Finally, the comparisons on the plasma consumed were similar to those of RBCs, and the differences among RS1 versus RS2 and RS2 versus RS3 were statistically significant (*P* < 0.001) but not between RS3 and RS4 (*P* = 0.305). All the data are shown in Fig. 2.

## Discussion

Blood transfusion is inevitable for many difficult surgeries, although there are numerous potential adverse blood transfusion reactions, including nonhemolytic fever reactions, allergic reactions, and hemolytic reactions [14]; at the same time, the inhibition of cellular immune function caused by blood transfusion increased the risk of nosocomial infection [15]; infusion of RBCs with different storage times is also closely related to patient prognosis [16–18]. It is also critical for infants with CHD because the ratio of extracorporeal circulation pipeline precharge to infants’ blood volume is larger, and a certain number of RBCs must be precharged to maintain a satisfactory Hct during extracorporeal circulation operation, which increases infants’ reliance on blood transfusion under CPB [19]. It has been shown that there is a significant correlation between the amount of blood transfusions and the prognosis of children with CHD [20, 21]. Therefore, it is extremely beneficial for patients to carry out intraoperative blood transfusions scientifically and reasonably, and the establishment of an ideal preoperative blood reserve system is the foundation for accomplishing the above objectives, which include effective blood protection techniques, correction of preoperative anemia, improvement of coagulation, reduction of intraoperative bleeding, autotransfusion, and reduction of heterotransfusion.

We studied the general characteristics of patients and the results of preoperative laboratory tests, as well as the factors influencing blood use, based on intraoperative blood transfusions of patients undergoing CHS under CPB in our hospital. The research revealed that the majority of patients undergoing CHS were under 2 years, particularly those aged 0–1 year, which is consistent with previous data [22]. Following the previous analysis, the chi-square test was employed as a single factor analysis to filter the significant variables, and binary logistic regression analysis was performed as a multivariate analysis. The results showed that age, weight, RACHS method, and preoperative hemoglobin were the influencing factors for transfusing RBCs; age, weight, and RACHS method were the influencing factors for platelets transfusion; and age, weight, RACHS method, APTT, and PT were the influential factors for plasma infusion. There was an interesting result in that platelet counts did not differ significantly between whether or not to transfuse platelets, which may be attributed to preoperative management, and we further analyzed the differences using the independent-samples *t* test, which similarly yielded no significance (shown in Additional file 1). In a study evaluating blood for coronary artery bypass surgery, Ian Welsby reported that sex was an influential factor [12], although the result was not the same as in this study. Following up on the previous findings, we compared the transfused dosages of RBCs, platelets, and plasma across RACHS methods. The 1550 cases collected in this study were from RACHS methods 1–4, with no data from methods 5–6. Some studies have shown that intraoperative hemorrhage is an influential factor in intraoperative blood transfusion [12, 23], which is associated with complex surgery. In this study, blood transfusion dosages increased as the RACHS grade increased. However, due to a lack of RACHS methods in 5–6 cases, it is unclear whether the dosage of these three blood components will continue to rise.

In the introduction, we discussed the fact that the actual amount of blood used in some surgeries is greater than the amount prepared. This phenomenon is most common in more complicated surgeries, and it is caused by a lack of assessment of the complexity of the surgery and uncontrollable changes during the procedure. In this study, we found that the RACHS method is a key factor affecting the volume of intraoperative blood transfused, and it could even be said to be the most important factor, so it should be considered first in clinical blood preparation. Especially for surgeries with higher RACHS grades, the blood reserve volume can be appropriately increased in clinical practice. The second question we should focus on is the comparison of restrictive and liberal blood transfusions during CHS. According to Matthias Redlin, blood transfusion in pediatric cardiac surgery determines postoperative morbidity by comparing mechanical ventilation, intensive care unit stay, and cardiopulmonary time in the no transfusion, postoperative transfusion only, and intraoperative transfusion groups [24], and these findings may encourage attending physicians to implement stringent blood-sparing approaches. Similar findings were also reported in another study in which RBCs transfusions were associated with prolonged mechanical ventilation in children with acute respiratory distress syndrome [23]. However, Jean A Ballweg announced that an overemphasis on the use of blood during CHS is not conducive to the postoperative mental development of children [13]. As a result, whether patients who receive CHS should perform a liberal or restrictive blood transfusion strategy is still not well known, a topic on which more technologists and cardiologists need to focus.

## Supplementary Information

**Additional file 1.** The comparison of platelet counts between platelets transfused and untransfused.

## Data Availability

The datasets used and/or analyzed during the present study are available from the corresponding author on reasonable request.
