# Supplementary figures and images for: The characteristics of blood transfusion and analysis of preoperative factors associated with intraoperative blood transfusion in congenital heart surgery: a case–control study
Source: J Cardiothorac Surg. 2022 Dec 24;17:337. doi: 10.1186/s13019-022-02068-2 (PMC9789642; doi:10.1186/s13019-022-02068-2)

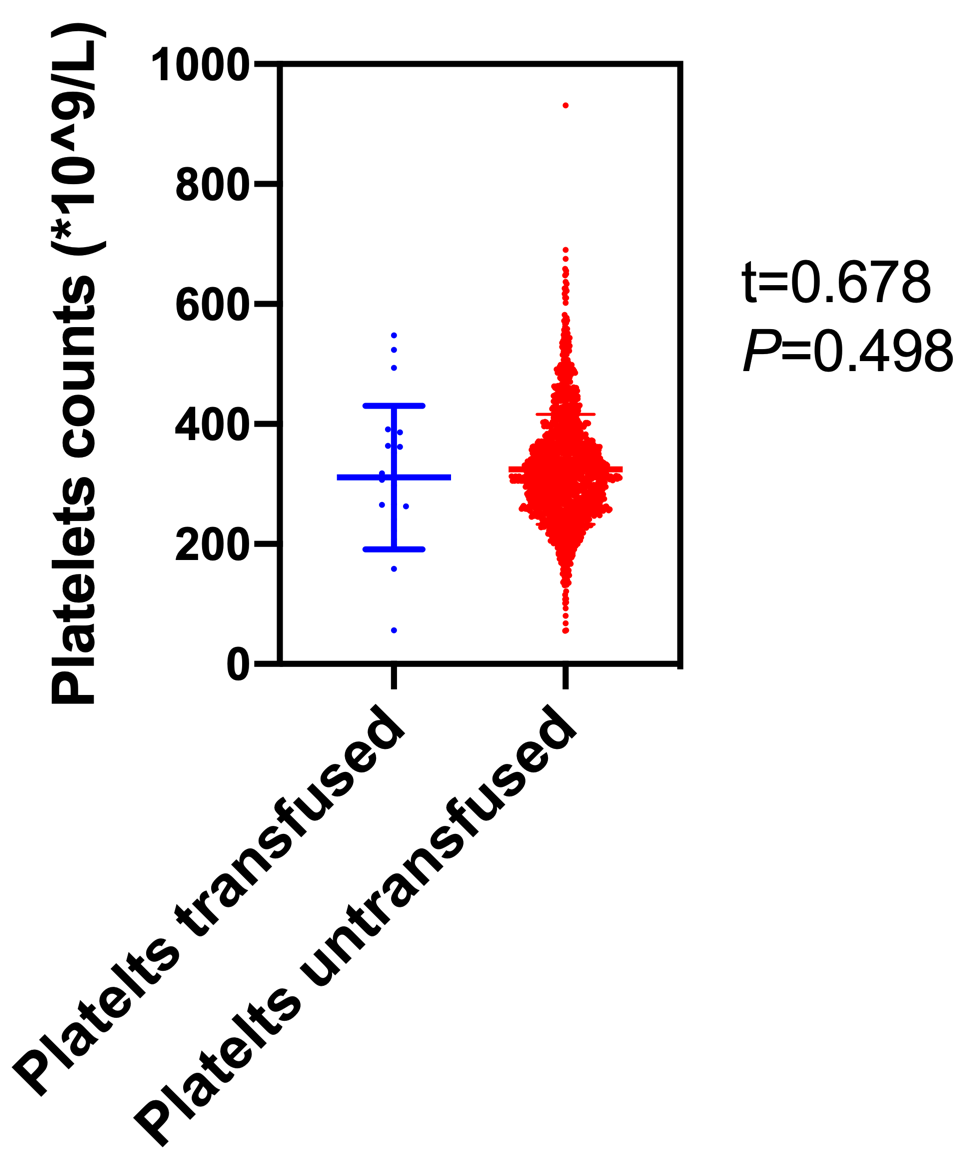

Supplement: Supplementary file 1 — Additional file 1. The comparison of platelet counts between platelets transfused and untransfused. [file 13019_2022_2068_MOESM1_ESM.tiff]
